# Supplementary material for: Role of Statins in the Primary Prevention of Atherosclerotic Cardiovascular Disease and Mortality in the Population with Mean Cholesterol in the Near-Optimal to Borderline High Range: A Systematic Review and Meta-Analysis
Source: Adv Prev Med. 2020 Nov 21;2020:6617905. doi: 10.1155/2020/6617905 (PMC7700057; doi:10.1155/2020/6617905)
Supplement: Supplementary Materials — There were four supplementary materials. The first one is the “search strategies for different electronic databases,” the second one is the “PRISMA checklist,” and the third one is Supplement Tables 1–5. Supplement Table 1: patient baseline characteristics and interventions used in the included trials. Supplement Table 2: study design and target population of the included trials. Supplement Table 3: sensitivity analysis stratified by the trial characteristics. Supplement Table 4: sensitivity analysis stratified for the type of population. Supplement Table 5: grade of evidence. The fourth Supplementary Material is Supplement Figures 1–9. Supplement Figure 1: meta-analysis of major coronary events. Supplement Figure 2: meta-analysis of composite cardiovascular outcome. Supplement Figure 3: meta-analysis of coronary heart disease mortality. Supplement Figure 4: meta-analysis of muscle-related adverse events. Supplement Figure 5: meta-analysis of incidence of other adverse events. Supplement Figure 6: meta-analysis of revascularizations. Supplement Figure 7: meta-analysis of angina. Supplement Figure 8: meta-analysis of hospitalizations for cardiovascular causes. Supplement Figure 9: funnel plot for composite cardiovascular outcomes. [file 6617905.f1.docx]

**Supplemental Material**

Supplementary Material 1: Search strategies for different electronic databases

| Electronic databases | Search Strategy |
| --- | --- |
| PubMed Central (PMC) | (((hydroxymethylglutaryl-coa reductase inhibitors[MeSH Terms]) OR (simvastatin[MeSH Terms]) OR (lovastatin[MeSH Terms]) OR (pravastatin[MeSH Terms]) OR (atorvastatin[MeSH Terms]) OR (cerivastatin[All Fields]) OR (fluvastatin[MeSH Terms]) OR (rosuvastatin calcium[MeSH Terms]) OR (pitavastatin[All Fields]) OR (Statin[All Fields])) AND ((coronary artery disease[MeSH Terms]) OR (heart diseases[MeSH Terms]) OR (coronary disease[MeSH Terms]) OR (cardiovascular diseases[MeSH Terms]) OR (myocardial infarction[MeSH Terms]) OR (cerebrovascular disorders[MeSH Terms]) OR (stroke[MeSH Terms]) OR (angina pectoris[MeSH Terms]) OR (ischemic attack, transient[MeSH Terms]) OR (mortality[MeSH Terms])) AND ((randomized controlled trials as topic[MeSH Terms]) OR (controlled clinical trials as topic[MeSH Terms]) OR (random allocation[MeSH Terms]) OR (randomly[All Fields]) OR (clinical trials as topic[MeSH Terms]) OR (placebos[MeSH Terms]) OR (primary prevention[MeSH Terms]) OR (cholesterol[MeSH Terms]) OR (cholesterol, ldl[MeSH Terms]))) |
| PubMed | (((hydroxymethylglutaryl-coa reductase inhibitors[MeSH Terms]) OR (simvastatin[MeSH Terms]) OR (lovastatin[MeSH Terms]) OR (pravastatin[MeSH Terms]) OR (atorvastatin[MeSH Terms]) OR (cerivastatin[All Fields]) OR (fluvastatin[MeSH Terms]) OR (rosuvastatin calcium[MeSH Terms]) OR (pitavastatin[All Fields]) OR (statin[Text Word])) AND ((coronary artery disease[MeSH Terms]) OR (heart diseases[MeSH Terms]) OR (coronary disease[MeSH Terms]) OR (cardiovascular diseases[MeSH Terms]) OR (myocardial infarction[MeSH Terms]) OR (cerebrovascular disorders[MeSH Terms]) OR (stroke[MeSH Terms]) OR (ischemic attack, transient[MeSH Terms]) OR (angina pectoris[MeSH Terms]) OR (mortality[MeSH Terms]) OR (primary prevention[MeSH Terms]) OR (cholesterol[MeSH Terms]) OR (cholesterol, ldl[MeSH Terms]) OR (placebos[MeSH Terms]))) |
| Cochrane Library | #1 MeSH descriptor: [Hydroxymethylglutaryl-CoA Reductase Inhibitors] explode all trees |
|  | #2 MeSH descriptor: [Simvastatin] explode all trees |
|  | #3 MeSH descriptor: [Lovastatin] explode all trees |
|  | #4 MeSH descriptor: [Pravastatin] explode all trees |
|  | #5 MeSH descriptor: [Atorvastatin] explode all trees |
|  | #6 MeSH descriptor: [Fluvastatin] explode all trees |
|  | #7 MeSH descriptor: [Rosuvastatin Calcium] explode all trees |
|  | #8 (cerivastatin) |
|  | #9 (pitavastatin) |
|  | #10 (statins) |
|  | #11 MeSH descriptor: [Coronary Artery Disease] explode all trees |
|  | #12 MeSH descriptor: [Coronary Disease] explode all trees |
|  | #13 MeSH descriptor: [Cardiovascular Diseases] explode all trees |
|  | #14 MeSH descriptor: [Heart Diseases] explode all trees |
|  | #15 MeSH descriptor: [Cardiovascular Abnormalities] explode all trees |
|  | #16 MeSH descriptor: [Myocardial Infarction] explode all trees |
|  | #17 MeSH descriptor: [Cerebrovascular Disorders] explode all trees |
|  | #18 MeSH descriptor: [Stroke] explode all trees |
|  | #19 MeSH descriptor: [Angina Pectoris] explode all trees |
|  | #20 MeSH descriptor: [Ischemic Attack, Transient] explode all trees |
|  | #21 MeSH descriptor: [Mortality] explode all trees |
|  | #22 MeSH descriptor: [Multicenter Studies as Topic] explode all trees |
|  | #23 MeSH descriptor: [Controlled Clinical Trials as Topic] explode all trees |
|  | #24 MeSH descriptor: [Randomized Controlled Trials as Topic] explode all trees |
|  | #25 MeSH descriptor: [Clinical Trials as Topic] explode all trees |
|  | #26 MeSH descriptor: [Random Allocation] explode all trees |
|  | #27 MeSH descriptor: [Placebos] explode all trees |
|  | #28 MeSH descriptor: [Primary Prevention] explode all trees |
|  | #29 MeSH descriptor: [Cholesterol] explode all trees |
|  | #30 MeSH descriptor: [Cholesterol, LDL] explode all trees |
|  | #31 ((# 1 OR #2 OR #3 OR #4 OR #5 OR #6 OR #7 OR #8 OR #9 OR #10) AND ( #11 OR #12 OR #13 OR #14 OR #15 OR #16 OR #17 OR #18 OR #19 OR #20 OR #21) AND ( #22 OR #23 OR #24 OR #25 OR #26 OR #27 OR #28 OR #29 OR #30)) with Publication Year from 1994 to 2020, in Trials |
| Google Scholar | With all of the words: Primary prevention, Statins, Hydroxymethylglutaryl CoA inhibitors, Cardiovascular disease events, Stroke, Myocardial infarction, angina pectoris, Mortality, Coronary artery disease, Randomized Controlled Trial, Clinical Trial, Cholesterol, LDL, Placebo  With the exact phrase: primary prevention  With at least one of the words: simvastatin, lovastatin, pravastatin, atorvastatin, cerivastatin, fluvastatin, rosuvastatin, pitavastatin, heart diseases, coronary disease, cerebrovascular disorders, cardiovascular disorders, cardiovascular abnormalities, Coronary deaths, transient ischemic attack, random allocation, randomly  Where my words occur: anywhere in the article  Return articles dated between: 1994-2020 |

Supplementary Material 2: PRISMA checklist

| **ection/topic** | **#** | **Checklist item** | **Reported on page #** |
| --- | --- | --- | --- |
| **TITLE** | | |  |
| Title | 1 | Identify the report as a systematic review, meta-analysis, or both. | 1 |
| **ABSTRACT** | | |  |
| Structured summary | 2 | Provide a structured summary including, as applicable: background; objectives; data sources; study eligibility criteria, participants, and interventions; study appraisal and synthesis methods; results; limitations; conclusions and implications of key findings; systematic review registration number. | 1, 2 |
| **INTRODUCTION** | | |  |
| Rationale | 3 | Describe the rationale for the review in the context of what is already known. | 2 |
| Objectives | 4 | Provide an explicit statement of questions being addressed with reference to participants, interventions, comparisons, outcomes, and study design (PICOS). | 3 |
| **METHODS** | | |  |
| Protocol and registration | 5 | Indicate if a review protocol exists, if and where it can be accessed (e.g., Web address), and, if available, provide registration information including registration number. | 3 |
| Eligibility criteria | 6 | Specify study characteristics (e.g., PICOS, length of follow-up) and report characteristics (e.g., years considered, language, publication status) used as criteria for eligibility, giving rationale. | 3, 4 |
| Information sources | 7 | Describe all information sources (e.g., databases with dates of coverage, contact with study authors to identify additional studies) in the search and date last searched. | 3 |
| Search | 8 | Present full electronic search strategy for at least one database, including any limits used, such that it could be repeated. | 3 |
| Study selection | 9 | State the process for selecting studies (i.e., screening, eligibility, included in systematic review, and, if applicable, included in the meta-analysis). | 3, 4 |
| Data collection process | 10 | Describe method of data extraction from reports (e.g., piloted forms, independently, in duplicate) and any processes for obtaining and confirming data from investigators. | 4 |
| Data items | 11 | List and define all variables for which data were sought (e.g., PICOS, funding sources) and any assumptions and simplifications made. | 4 |
| Risk of bias in individual studies | 12 | Describe methods used for assessing risk of bias of individual studies (including specification of whether this was done at the study or outcome level), and how this information is to be used in any data synthesis. | 4 |
| Summary measures | 13 | State the principal summary measures (e.g., risk ratio, difference in means). | 4, 5 |
| Synthesis of results | 14 | Describe the methods of handling data and combining results of studies, if done, including measures of consistency (e.g., I^2^) for each meta-analysis. | 4, 5 |

| **Section/topic** | **#** | **Checklist item** | **Reported on page #** |
| --- | --- | --- | --- |
| Risk of bias across studies | 15 | Specify any assessment of risk of bias that may affect the cumulative evidence (e.g., publication bias, selective reporting within studies). | 5 |
| Additional analyses | 16 | Describe methods of additional analyses (e.g., sensitivity or subgroup analyses, meta-regression), if done, indicating which were pre-specified. | 4, 5 |
| **RESULTS** | | |  |
| Study selection | 17 | Give numbers of studies screened, assessed for eligibility, and included in the review, with reasons for exclusions at each stage, ideally with a flow diagram. | 5, 6 |
| Study characteristics | 18 | For each study, present characteristics for which data were extracted (e.g., study size, PICOS, follow-up period) and provide the citations. | 6, 7 |
| Risk of bias within studies | 19 | Present data on risk of bias of each study and, if available, any outcome level assessment (see item 12). | 7, 14, 15 |
| Results of individual studies | 20 | For all outcomes considered (benefits or harms), present, for each study: (a) simple summary data for each intervention group (b) effect estimates and confidence intervals, ideally with a forest plot. | 7-13 |
| Synthesis of results | 21 | Present results of each meta-analysis done, including confidence intervals and measures of consistency. | 7-13 |
| Risk of bias across studies | 22 | Present results of any assessment of risk of bias across studies (see Item 15). | 14 |
| Additional analysis | 23 | Give results of additional analyses, if done (e.g., sensitivity or subgroup analyses, meta-regression [see Item 16]). | 13, 14 |
| **DISCUSSION** | | |  |
| Summary of evidence | 24 | Summarize the main findings including the strength of evidence for each main outcome; consider their relevance to key groups (e.g., healthcare providers, users, and policy makers). | 15-18 |
| Limitations | 25 | Discuss limitations at study and outcome level (e.g., risk of bias), and at review-level (e.g., incomplete retrieval of identified research, reporting bias). | 18 |
| Conclusions | 26 | Provide a general interpretation of the results in the context of other evidence, and implications for future research. | 18 |
| **FUNDING** | | |  |
| Funding | 27 | Describe sources of funding for the systematic review and other support (e.g., supply of data); role of funders for the systematic review. | 19 |

Supplementary Material 3: Supplement Table 1-5

| **Study** | **Statin/ control (No.)** | **Follow-up duration in years (mean)** | **Prior CVD (%)** | **Participant characteristics** | | | | | | | **Medicine dose (mg) versus (vs) control** | **Mean lipid level, (mg/dl) TC, LDL-C** | **Family H/O early CHD (%)** |
| --- | --- | --- | --- | --- | --- | --- | --- | --- | --- | --- | --- | --- | --- |
|  |  |  |  | Mean age (years) | Diabetes mellitus (%) | Current smokers (%) | Mean, SBP/ DBP, mm of Hg | Race (%) | Mean BMI, Kg/m^2^ | Women (%) |  |  |  |
| AFCAPS/TexCAPS, 1998 | 3304/ 3301 | 5.2 | < 1 | 58 | 6.1 | 12.5 | 138/78 | W: 89; B: 3; H: 7 | 26.5 | 15 | Lovastatin vs matching placebo; 20 titrated 40 if LDL-C>110 | TC:221; LDL-C: 150 Median | 15.5 |
| ALLHAT-LLT*, 2017 | 1467/ 1400 | 4.8 | 0 | 71.2 | 51 | 22.3 | 147.4/83.4 | W: 57.1; B: 37.9; O: 4.8 | 29.5 | 49.3 | Pravastatin 40 vs usual care | TC: 225.6; LDL-C: 147.6 | NA |
| Beishuizen et al, 2004 | 125/ 125 | 2 | 0 | 59 | 100 | 24 | HTN: 51% | W: 68; IA: 19 O: 13 | 31 | 53 | Cerivastatin 0.4 then Simvastatin 20 vs Placebo | TC: 215; LDL-C: 135 | NA |
| CARDS, 2004 | 1428/ 1410 | 3.9 (median) | < 1 | 62 | 100 | 23 | 144/83 | W: 95 | 29 | 32 | Atorvastatin 10 vs Placebo | TC: 207; LDL-C: 118 | NA |
| HOPE-3, 2016 | 6361/ 6344 | 5.6 (median) | 0 | 65.7 | 5.8 | 28 | 138/82 | H: 27.5; A: 49; W: 20; B: 1.8 | 27.1 | 46 | Rosuvastatin 10 vs Placebo | TC: 201; LDL-C: 128 | 26 |
| HYRIM, 2004 | 142/ 143 | 4 | 0 | 57 | NA | 18.3 | 141/88 | NA | 29.2 | NA | Fluvastatin 40 vs Placebo | TC: 230; LDL-C: 150 | NA |
| JUPITER, 2008 | 8901/ 8901 | 1.9 (median) | 0 | 66 (media-n) | Metabolic Syndrome (42%) | 16 | 134/80 | W: 71; B: 13; H: 13 | 28 (media-n) | 39 | Rosuvastatin 20 vs Placebo | TC: 185.5; LDL-C: 108 Median | 12 |
| MEGA, 2006 | 3866/ 3966 | 5.3 | < 1 | 58.3 | 21 | 20.5 | HTN: 42% | NA | 23.8 | 68.5 | Diet + pravastatin vs diet; 10 titrated 20 if TC did not decrease ≤ 220 mg/dl | TC: 242.5; LDL-C: 156.6 | NA |
| PREVEND IT, 2004 | 433/431 | 3.8 | 3.3 | 52 | 3 | 40 | 131/77 | W: 96 | 26 | 35 | Pravastatin 40 vs Placebo | TC: 224; LDL-C: 157 (median) | NA |
| PROSPER*, 2002 | 1585/ 1654 | 3.2 | 0 | 75 | 12.2 | 33.4 | 156.6/85.2 | NA | 27 | 58 | Pravastatin 40 vs Placebo | TC: 220; LDL-C: 147 | NA |
| TRACE RA, 2019 | 1504/ 1498 | 2.51 (median) | 0 | 61 | 0 | 16.5 | HTN: 22.5% | W: 98 | 26.6 (media-n) | 74 | Atorvastatin 40 vs Placebo | TC: 207; LDL-C: 124 (median) | 21 |

Supplement Table 1. Patient baseline characteristics and interventions used in the included trials

A, Asian; B, black; BMI, body mass index; CVD, cardiovascular disease; CHD, coronary heart disease; DBP, diastolic blood pressure; H, Hispanic; H/O, history of; HTN, hypertension; IA, Indo-Asian; LDL-C, low density lipoprotein-cholesterol; NA, not available; O, other ethnic groups; SBP, systolic blood pressure; TC, total cholesterol; W, white

AFCAPS/TexCAPS, Air Force/Texas Coronary Atherosclerosis Prevention Study; ALLHAT-LLT, Antihypertensive and Lipid-Lowering Treatment to Prevent Heart Attack; CARDS, Collaborative Atorvastatin Diabetes Study; HOPE–3, Heart Outcomes Prevention Evaluation; HYRIM, Hypertension High Risk Management trial; JUPITER, Justification for the Use of Statins in Prevention: An Intervention Trial Evaluating Rosuvastatin; MEGA, Management of Elevated Cholesterol in the Primary Prevention Group of Adult Japanese; PREVEND IT, the Prevention of REnal and Vascular ENdstage Disease Intervention Trial; PROSPER, PROspective Study of Pravastatin in the Elderly at Risk; TRACE RA, Trial of Atorvastatin for the Primary Prevention of Cardiovascular Events in Patients with Rheumatoid Arthritis.

*Data analyzed from the primary prevention arm of the clinical trial, free from cardiovascular disease at baseline

| **Study** | **Target Population** | **Study Design** | **Statistical Analysis calculation** |
| --- | --- | --- | --- |
| AFCAPS/TexCAPS, 1998 | Men and women with average TC and LDL-C and below-average HDL-C without CVD | Randomized, double-blind, placebo-controlled primary prevention trial | Cox proportional hazards regression model |
| ALLHAT-LLT*, 2017 | 2867 older adults ≥ 65 Y without atherosclerotic cardiovascular disease as a subset of main study ALLHAT-LLT | Randomized, non-blinded, large simple trial | Cox proportional hazards model |
| Beishuizen et al, 2004 | Participants aged 30-80 years without CVD but Type 2 DM for at least one year | Randomized, placebo-controlled, double-blind clinical trial | Clinical events as proportion & evaluate using χ2 or Fisher’s test |
| CARDS, 2004 | Age 40-75 Y with Type 2 DM with at least one: retinopathy, albuminuria, smoker, or HTN. Participants had no CVD, LDL-C ≤ 160 mg/dl | A multicenter randomized placebo-controlled trial | Cox regression survival analysis |
| HOPE-3, 2016 | Men ≥ 55 Y and women ≥ 65 Y without CVD had at least one: elevated waist-to-hip ratio, H/O low HDL-C, current tobacco use, dysglycemia, family H/O premature CHD, and mild renal dysfunction. | A pragmatic, multicenter, long-term, international, double-blind, randomized, placebo-controlled trial based on 2-by-2 factorial design | Cox proportional hazards model |
| HYRIM, 2004 | Men 40-74 Y with HTN, without CVD, and TC 174-309 mg/dl, TG < 398 mg/dl, BMI 25-35 kg/m2, and sedentary lifestyle. | Randomized, placebo-controlled, 2-by-2 factorial design | Secondary outcome variables as no. of CVD events |
| JUPITER, 2008 | Men≥ 50 Y and women ≥ 60 Y without CVD and screening LDL-C < 130 mg/dl and high-sensitivity C-reactive protein ≥ 2 mg/L. | Randomized, double-blind, placebo-controlled, multicenter trial | Cox proportional hazards model |
| MEGA, 2006 | Men and postmenopausal women aged 40-70 Y without any H/O CHD or stroke. Participants with familial hypercholesterolemia were excluded. | Prospective randomized, open-labeled, blinded-endpoint study | Cox’s proportional hazards model |
| PREVEND IT, 2004 | Participants had persistent microalbuminuria, blood pressure < 160/100 mm of Hg, TC < 309 mg/dl or < 193 mg/dl in case of previous MI | Single-center, double-blind, randomized, placebo-controlled trial with a 2-by-2 factorial design | Two-way ANOVA; HR or RR with 95% CI |
| PROSPER*, 2002 | 3239 participants without cardiovascular disease as a primary preventive subgroup of the main study | Randomized placebo-controlled and blinded trial | HR and P values from the Cox model analyses |
| TRACE RA, 2019 | Rheumatoid arthritis (RA) patients > 50 years of age and had disease >10 years duration. Those with CVD, DM, myopathy, taking statins were excluded | Randomized, double-blind, placebo-controlled, multicenter clinical trial | Cox regression models |

Supplement Table 2. Study design and target population of the included trials

ANOVA, analysis of variance; BMI, body mass index; CVD, cardiovascular disease; CI, confidence interval; CHD, coronary heart disease; DM, diabetes mellitus; HR, hazard ratio; HDL-C, high density lipoprotein-cholesterol; H/O, history of; HTN, hypertension; MI, myocardial infarction; RR, risk ratio; TC, total cholesterol; TG, triglyceride; Y, year

AFCAPS/TexCAPS, Air Force/Texas Coronary Atherosclerosis Prevention Study; ALLHAT-LLT, Antihypertensive and Lipid-Lowering Treatment to Prevent Heart Attack; CARDS, Collaborative Atorvastatin Diabetes Study; HOPE–3, Heart Outcomes Prevention Evaluation; HYRIM, Hypertension High Risk Management trial; JUPITER, Justification for the Use of Statins in Prevention: An Intervention Trial Evaluating Rosuvastatin; MEGA, Management of Elevated Cholesterol in the Primary Prevention Group of Adult Japanese; PREVEND IT, the Prevention of REnal and Vascular ENdstage Disease Intervention Trial; PROSPER, PROspective Study of Pravastatin in the Elderly at Risk; TRACE RA, Trial of Atorvastatin for the Primary Prevention of Cardiovascular Events in Patients with Rheumatoid Arthritis.

*Data analyzed from the primary prevention arm of the clinical trial, free from cardiovascular disease at baseline

| **Analysis** | **Composite Cardiovascular Outcomes** | **Myocardial Infarction** | **Major Cerebrovascular Events** | **Major Coronary Events** | **Cardiovascular Mortality** | **All-Cause Mortality** | **Revascularizations** | **CHD Mortality** | **Angina** |
| --- | --- | --- | --- | --- | --- | --- | --- | --- | --- |
| **Trials with a study population less than 3000** | | | | | | | | | |
| RR with I^2^ | 0.71 [0.53, 0.94]  I^2^ = 40% | 0.53 [0.35, 0.81]  I^2^ not applicable | 0.88 [0.49, 1.56]  I^2^ = 65% | 0.75 [0.61, 0.92]  I^2^ = 0% | 0.94 [0.65, 1.35]  I^2^ = 27% | 0.95 [0.72, 1.24]  I^2^ = 36% | 0.70 [0.42, 1.17]  I^2^ not applicable | 0.87 [0.63, 1.21]  I^2^ = 0% | 0.77 [0.29, 2.06]  I^2^ not applicable |
| No. of trials | 5 | 1 | 3 | 2 | 3 | 5 | 1 | 2 | 1 |
| **Trials with a study population greater than 3000** | | | | | | | | | |
| RR with I^2^ | 0.70 [0.60, 0.84]  I^2^ = 68% | 0.57 [0.47, 0.69]  I^2^ = 0% | 0.75 [0.59, 0.94]  I^2^ = 39% | 0.63 [0.50, 0.81]  I^2^ = 55% | 0.85 [0.71, 1.01]  I^2^ = 0% | 0.90 [0.82, 0.98]  I^2^ = 0% | 0.64 [0.56, 0.74]  I^2^ = 0% | 0.81 [0.43, 1.54]  I^2^ = 0% | 0.76 [0.63, 0.93]  I^2^ = 0% |
| No. of trials | 6 | 5 | 6 | 6 | 5 | 6 | 6 | 3 | 4 |
| Test for subgroup difference | Chi² = 0.00, df = 1 (P = 0.99), I² = 0% | Chi² = 0.07, df = 1 (P = 0.80), I² = 0% | Chi² = 0.26, df = 1 (P = 0.61), I² = 0% | Chi² = 1.14, df = 1 (P = 0.29), I² = 12.0% | Chi² = 0.27, df = 1 (P = 0.60), I² = 0% | Chi² = 0.12, df = 1 (P = 0.73), I² = 0% | Chi² = 0.09, df = 1 (P = 0.77), I² = 0% | Chi² = 0.04, df = 1 (P = 0.84), I² = 0% | Chi² = 0.00, df = 1 (P = 0.99), I² = 0% |
| **Trials with adequate randomization** | | | | | | | | | |
| RR with I^2^ | 0.74 [0.64, 0.85]  I^2^ = 53% | 0.55 [0.44, 0.67]  I^2^ = 0% | 0.77 [0.61, 0.98]  I^2^ = 53% | 0.68 [0.56, 0.83]  I^2^ = 46% | 0.91 [0.79, 1.06]  I^2^ = 0% | 0.91 [0.81, 1.02]  I^2^ = 44% | 0.63 [0.54, 0.75]  I^2^ = 0% | 0.88 [0.65, 1.21]  I^2^ = 0% | 0.80 [0.64, 1.02]  I^2^ = 0% |
| No. of trials | 8 | 5 | 8 | 7 | 7 | 8 | 6 | 4 | 4 |
| **Trials with inadequate randomization** | | | | | | | | | |
| RR with I^2^ | 0.53 [0.28, 0.99]  I^2^=58% | 0.59 [0.43, 0.83]  I^2^ not applicable | 0.82 [0.41, 1.67]  I^2^ not applicable | 0.60 [0.43, 0.83]  I^2^ not applicable | 0.68 [0.37, I^2^ not applicable e | 1.00 [0.75, 1.34]  I^2^ = 0% | 0.67 [0.53, 0.86]  I^2^ not applicable | 0.73 [0.34, 1.59]  I^2^ not applicable | 0.69 [0.50, 0.95]  I^2^ not applicable |
| No. of trials | 3 | 1 | 1 | 1 | 1 | 3 | 1 | 1 | 1 |
| Test for subgroup difference | Chi² = 0.98, df = 1 (P = 0.32), I² = 0% | Chi² = 0.22, df = 1 (P = 0.64), I² = 0% | Chi² = 0.03, df = 1 (P = 0.87), I² = 0% | Chi² = 0.45, df = 1 (P = 0.50), I² = 0% | Chi² = 0.83, df = 1 (P = 0.36), I² = 0% | Chi² = 0.34, df = 1 (P = 0.56), I² = 0% | Chi² = 0.17, df = 1 (P = 0.68), I² = 0% | Chi² = 0.19, df = 1 (P = 0.66), I² = 0% | Chi² = 0.57, df = 1 (P = 0.45), I² = 0% |
| **Trials with study population unblinded** | | | | | | | | | |
| RR with I^2^ | 0.75 [0.62, 0.90]  I^2^ = 0% | 0.53 [0.3, 0.95]  I^2^ not applicable | 0.94 [0.74, 1.20]  I^2^ not applicable | 0.71 [0.49, 1.02]  I^2^ = 39% | 0.93 [0.56, 1.56]  I^2^ = 49% | 0.92 [0.59, 1.46]  I^2^ = 83% | 0.61 [0.41, 0.90]  I^2^ not applicable | 0.92 [0.63, 1.35]  I^2^ = 0% | 0.83 [0.56, 1.22]  I^2^ not applicable |
| No. of trials | 2 | 1 | 2 | 2 | 2 | 2 | 1 | 2 | 1 |
| **Trials with study population blinded** | | | | | | | | | |
| RR with I^2^ | 0.70 [0.59, 0.83]  I^2^ = 63% | 0.56 [0.47, 0.68] I^2^ = 0% | 0.71 [0.54, 0.94]  I^2^ = 47% | 0.65 [0.52, 0.81]  I^2^ = 52% | 0.85 [0.71, 1.03]  I^2^ = 0% | 0.90 [0.82, 0.98]  I^2^ = 0% | 0.65 [0.57, 0.75]  I^2^ = 0% | 0.78 [0.49, 1.23]  I^2^ = 0% | 0.74 [0.60, 0.92]  I^2^ = 0% |
| No. of trials | 9 | 5 | 7 | 6 | 6 | 9 | 6 | 3 | 4 |
| Test for subgroup difference | Chi² = 0.22, df = 1 (P = 0.64), I² = 0% | Chi² = 0.05, df = 1 (P = 0.83), I² = 0% | Chi² = 2.16, df = 1 (P = 0.14), I² = 53.8% | Chi² = 0.15, df = 1 (P = 0.70), I² = 0% | Chi² = 0.21, df = 1 (P = 0.65), I² = 0% | Chi² = 0.01, df = 1 (P = 0.91), I² = 0% | Chi² = 0.12, df = 1 (P = 0.73), I² = 0% | Chi² = 0.31, df = 1 (P = 0.58), I² = 0% | Chi² = 0.23, df = 1 (P = 0.63), I² = 0% |
| **Trials with a study population average of LDL-C in borderline-high range** | | | | | | | | | |
| RR with I^2^ | 0.74 [0.61, 0.91]  I^2^ = 58% | 0.58 [0.44, 0.77]  I^2^ = 0% | 0.97 [0.80, 1.18]  I^2^ = 0% | 0.74 [0.60, 0.92]  I^2^ = 49% | 0.93 [0.69, 1.24]  I^2^ = 14% | 1.00 [0.88, 1.14]  I^2^ = 10% | 0.68 [0.57, 0.82]  I^2^ = 0% | 0.88 [0.63, 1.24]  I^2^ = 0% | 0.74 [0.58, 0.95]  I^2^ = 0% |
| No. of trials | 7 | 2 | 5 | 4 | 4 | 7 | 3 | 3 | 2 |
| **Trials with a study population average of LDL-C in near-optimal range** | | | | | | | | | |
| RR with I^2^ | 0.67 [0.56, 0.79]  I^2^=44% | 0.55 [0.44, 0.69]  I^2^ = 0% | 0.60 [0.48, 0.75]  I^2^ = 0% | 0.59 [0.47, 0.73]  I^2^ = 0% | 0.85 [0.71, 1.03]  I^2^ = 0% | 0.87 [0.78, 0.96]  I^2^ = 0% | 0.61 [0.50, 0.74]  I^2^ = 0% | 0.80 [0.46, 1.41]  I^2^ = 0% | 0.79 [0.59, 1.06]  I^2^ = 0% |
| No. of trials | 4 | 4 | 4 | 4 | 4 | 4 | 4 | 2 | 3 |
| Test for subgroup difference | Chi² = 0.67, df = 1 (P = 0.41), I² = 0% | Chi² = 0.10, df = 1 (P = 0.76), I² = 0% | Chi² = 10.20, df = 1 (P = 0.001), I² = 90.2% | Chi² = 2.32, df = 1 (P = 0.13), I² = 56.8% | Chi² = 0.77, df = 1 (P = 0.38), I² = 0% | Chi² = 3.05, df = 1 (P = 0.08), I² = 67.2% | Chi² = 0.70, df = 1 (P = 0.40), I² = 0% | Chi² = 0.08, df = 1 (P = 0.78), I² = 0% | Chi² = 0.09, df = 1 (P = 0.76), I² = 0% |
| **Trials with a mean follow up greater than 3.5 years** | | | | | | | | | |
| RR with I^2^ | 0.73 [0.66, 0.80]  I^2^=0% | 0.59 [0.48, 0.72]  I^2^ = 0% | 0.81 [0.65, 1.01]  I^2^ = 29% | 0.68 [0.59, 0.79]  I^2^ = 0% | 0.90 [0.77, 1.06]  I^2^ = 3% | 0.94 [0.81, 1.08]  I^2^ = 39% | 0.67 [0.56, 0.79]  I^2^ = 0% | 0.85 [0.63, 1.14]  I^2^ = 0% | 0.78 [0.64, 0.96]  I^2^ = 0% |
| No. of trials | 7 | 4 | 6 | 5 | 6 | 7 | 4 | 4 | 4 |
| **Trials with a mean follow up less than 3.5 years** | | | | | | | | | |
| RR with I^2^ | 0.63 [0.41, 0.97]  I^2^ = 84% | 0.48 [0.33, 0.69]  I^2^ = 0% | 0.65 [0.35, 1.23]  I^2^ = 74% | 0.63 [0.38, 1.06]  I^2^ = 77% | 0.81 [0.52, 1.27]  I^2^ = 0% | 0.88 [0.77, 1.00]  I^2^ = 0% | 0.67 [0.56, 0.79]  I^2^ = 18% | 1.32 [0.30, 5.92]  I^2^ not applicable | 0.59 [0.32, 1.10]  I^2^ not applicable |
| No. of trials | 4 | 2 | 3 | 3 | 2 | 4 | 3 | 1 | 1 |
| Test for subgroup difference | Chi² = 0.37, df = 1 (P = 0.55), I² = 0% | Chi² = 0.97, df = 1 (P = 0.32), I² = 0% | Chi² = 0.39, df = 1 (P = 0.53), I² = 0% | Chi² = 0.07, df = 1 (P = 0.79), I² = 0% | Chi² = 0.19, df = 1 (P = 0.66), I² = 0% | Chi² = 0.45, df = 1 (P = 0.50), I² = 0% | Chi² = 0.17, df = 1 (P = 0.68), I² = 0% | Chi² = 0.34, df = 1 (P = 0.56), I² = 0% | Chi² = 0.71, df = 1 (P = 0.40), I² = 0% |

Supplement Table 3. Sensitivity analysis stratified for the trial characteristics

| **Analysis** | **Composite Cardiovascular Outcomes** | **Myocardial Infarction** | **Major Cerebrovascular Events** | **Major Coronary Events** | **Cardiovascular Mortality** | **All-Cause Mortality** | **Revascularizations** | **CHD Mortality** | **Angina** |
| --- | --- | --- | --- | --- | --- | --- | --- | --- | --- |
| **All trials** | | | | | | | | | |
| RR (95% CI) | 0.71 [0.62, 0.82]  I^2^ = 55% | 0.56 [0.47, 0.67]  I^2^ = 0% | 0.78 [0.63, 0.96]  I^2^ = 47% | 0.67 [0.57, 0.80]  I^2^ = 44% | 0.90 [0.78, 1.04]  I^2^ = 0% | 0.92 [0.83, 1.02]  I^2^ = 25% | 0.65 [0.57, 0.74]  I^2^ = 0% | 0.86 [0.64, 1.15]  I^2^ = 0% | 0.76 [0.63, 0.92]  I^2^ = 0% |
| ARD (95% CI) | -1.239068[-1.642566, -0.835569] | -0.572599 [-0.865661, -0.279536] | -0.303956 [-0.451504, -0.156408] | -0.577777 [-0.862353, -0.293201] | -0.133922 [-0.260596, -0.007247] | -0.397156 [-0.667986, -0.126326] | -0.609761 [-0.816941, -0.40258] | -0.037307 [-0.15827, 0.083657] | -0.189295 [0.384342, 0.005752] |
| No. of Trials | 11 | 6 | 9 | 8 | 8 | 11 | 7 | 5 | 5 |
| **Trials/subgroup of trials with all participants having diabetes mellitus** | | | | | | | | | |
| RR (95% CI) | 0.45 [0.19, 1.04]  I^2^ = 56% | 0.53 [0.35, 0.81]  I^2^ not applicable | 0.59 [0.39, 0.89]  I^2^ = 0% | 0.65 [0.45, 0.95]  I^2^ not applicable | 0.65 [0.36, 1.15]  I^2^ not applicable | 0.70 [0.53, 0.92]  I^2^ = 0% | 0.70 [0.42, 1.17]  I^2^ not applicable | 0.74 [0.40, 1.36]  I^2^ not applicable | 0.77 [0.29, 2.06]  I^2^ not applicable |
| ARD (95% CI) | -5.393243 [-11.888009, 1.101522] | -2.015317 [-3.332496, -0.698137] | -1.063926 [-1.88759, -0.240263] | -1.598725 [-3.007163, -0.190286] | -0.726205 [-1.675934, 0.223523] | -1.413544 [-2.477416, -0.349672] | -0.730675 [-1.772613, 0.311262] | -0.441623 [-1.330811, 0.447564] | -0.148102 [-0.699474, 0.403271] |
| No. of Trials | 3 | 1 | 2 | 1 | 1 | 3 | 1 | 1 | 1 |
| Test for subgroup differences compared with all trials | Chi² = 1.14, df = 1 (P = 0.29), I²= 12.2% | Chi² = 0.05, df = 1 (P = 0.83), I² = 0% | Chi² = 1.35, df = 1 (P = 0.25), I² = 25.7% | Chi² = 0.02, df = 1 (P = 0.89), I² = 0% | Chi² = 1.17, df = 1 (P = 0.28), I² = 14.4% | Chi² = 3.41, df = 1 (P = 0.06), I² = 70.7% | Chi² = 0.07, df = 1 (P = 0.78), I² = 0% | Chi² = 0.19, df = 1 (P = 0.66), I² = 0% | Chi² = 0.00, df = 1 (P = 0.99), I² = 0% |
| **Trials/subgroup of trials with all participants having increased risk of cardiovascular diseases** | | | | | | | | | |
| RR (95% CI) | 0.66 [0.52, 0.82]  I^2^ = 48% | 0.48 [0.33, 0.69]  I^2^ = 0% | 0.50 [0.33, 0.74]  I^2^ = 0% | 0.48 [0.33, 0.69]  I^2^ = 0% | 0.81 [0.52, 1.27]  I^2^ = 0% | 0.81 [0.68, 0.97]  I^2^ = 0% | 0.54 [0.41, 0.72]  I^2^ = 0% | 1.33 [0.30, 5.92]  I^2^ not applicable | 0.59 [0.32, 1.10]  I^2^ = Not applicable |
| ARD (95% CI) | -1.15923 [-1.530932, -0.787528] | -0.431393 [-0.640469, -0.222318] | -0.34501 [-0.534268, -0.155753] | -0.431393 [-0.640469, -0.222318] | -0.077935 [-0.243971, 0.0881] | -0.470722 [-0.882325, -0.059119] | -0.618136 [-0.895253, -0.341019] | 0.06569 [-0.279277, 0.410658] | -0.123582 [-0.267788, 0.020625] |
| No. of Trials | 3 | 2 | 2 | 2 | 2 | 2 | 2 | 1 | 1 [18] |
| Test for subgroup differences compared with all trials | Chi² = 0.35, df = 1 (P = 0.55), I² = 0% | Chi² = 0.40, df = 1 (P = 0.53), I² = 0% | Chi² = 3.69, df = 1 (P = 0.05), I² = 72.9% | Chi² = 2.76, df = 1 (P = 0.10), I² = 63.7% | Chi² = 0.16, df = 1 (P = 0.69), I² = 0% | Chi² = 1.49, df = 1 (P = 0.22), I² = 32.9% | Chi² = 1.23, df = 1 (P = 0.27), I² = 18.6% | Chi² = 0.31, df = 1 (P = 0.58), I² = 0% | Chi² = 0.58, df = 1 (P = 0.44), I² = 0% |
| **Trials/subgroups of trials with all participants having LDL-C less than/equal to 160** | | | | | | | | | |
| RR (95% CI) | 0.65 [0.54, 0.77]  I^2^ = 53% | 0.49 [0.37, 0.67]  I^2^ = 0% | 0.52 [0.38, 0.72]  I^2^ = 0% | 0.55 [0.39, 0.79]  I^2^ = 35% | 0.72 [0.50, 1.05]  I^2^ = 0% | 0.78 [0.67, 0.92]  I^2^ = 0% | 0.58 [0.45, 0.74]  I^2^ = 0% | 0.74 [0.40, 1.36]  Not applicable | 0.64 [0.38, 1.08]  I^2^ = 0% |
| ARD (95% CI) | -1.284424 [-1.663295, -0.905552] | -1.122165 [-3.010404, 0.766074] | -0.703334 [-1.707101, 0.300432] | -0.871271 [-2.283694, 0.541151] | -0.273616 [-0.923958, 0.376726] | -0.751702 [-1.555881, 0.052477] | -0.678712 [-0.976737, -0.380687] | -0.441623 [-1.330811, 0.447564] | -0.125151 [-0.264665, 0.014362] |
| No. of Trials | 3 | 2 | 2 | 2 | 2 | 2 | 2 | 1 | 2 |
| Test for subgroup differences compared with all trials | Chi² = 0.72, df = 1 (P = 0.40), I² = 0% | Chi² = 0.53, df = 1 (P = 0.47), I² = 0% | Chi² = 3.96, df = 1 (P = 0.05), I² = 74.8% | Chi² = 0.96, df = 1 (P = 0.33), I² = 0% | Chi² = 1.12, df = 1 (P = 0.29), I² = 10.4% | Chi² = 2.81, df = 1 (P = 0.09), I² = 64.5% | Chi² = 0.65, df = 1 (P = 0.42), I² = 0% | Chi² = 0.19, df = 1 (P = 0.66), I² = 0% | Chi² = 0.40, df = 1 (P = 0.53), I² = 0% |

Supplement Table 4. Sensitivity analysis stratified for the type of population

| **Outcomes** | **№ of participants  (studies) Follow up** | **Certainty of the evidence (GRADE)** | **Relative effect (95% CI)** | **Anticipated absolute effects** | |
| --- | --- | --- | --- | --- | --- |
|  |  |  |  | **Risk with Control** | **Risk difference with Statins** |
| Composite Cardiovascular Outcomes | 58504 (11 RCTs) | ⨁⨁⨁◯ MODERATE ^a^ | **RR 0.71** (0.62 to 0.82) | 45 per 1,000 | **13 fewer per 1,000** (17 fewer to 8 fewer) |
| Myocardial Infarction | 50784 (6 RCTs) | ⨁⨁⨁⨁ HIGH | **RR 0.56** (0.47 to 0.67) | 14 per 1,000 | **6 fewer per 1,000** (7 fewer to 4 fewer) |
| Major Cerebrovascular Events | 57754 (9 RCTs) | ⨁⨁⨁⨁ HIGH | **RR 0.78** (0.63 to 0.96) | 14 per 1,000 | **3 fewer per 1,000** (5 fewer to 1 fewer) |
| Major Coronary Events | 56890 (8 RCTs) | ⨁⨁⨁⨁ HIGH | **RR 0.67** (0.57 to 0.80) | 22 per 1,000 | **7 fewer per 1,000** (9 fewer to 4 fewer) |
| Cardiovascular Mortality | 54515 (8 RCTs) | ⨁⨁⨁◯ MODERATE ^b^ | **RR 0.90** (0.78 to 1.04) | 14 per 1,000 | **1 fewer per 1,000** (3 fewer to 1 more) |
| All-Cause Mortality | 58504 (11 RCTs) | ⨁⨁⨁◯ MODERATE ^a^ | **RR 0.92** (0.83 to 1.02) | 43 per 1,000 | **3 fewer per 1,000** (7 fewer to 1 more) |
| Revascularization | 54023 (7 RCTs) | ⨁⨁⨁⨁ HIGH | **RR 0.65** (0.57 to 0.74) | 20 per 1,000 | **7 fewer per 1,000** (8 fewer to 5 fewer) |
| CHD Mortality | 23144 (5 RCTs) | ⨁⨁⨁◯ MODERATE ^c^ | **RR 0.86** (0.64 to 1.15) | 8 per 1,000 | **1 fewer per 1,000** (3 fewer to 1 more) |
| Angina | 47782 (5 RCTs) | ⨁⨁⨁◯ MODERATE ^d^ | **RR 0.76** (0.63 to 0.92) | 10 per 1,000 | **2 fewer per 1,000** (4 fewer to 1 fewer) |
| Serious Adverse Events | 42952 (5 RCTs) | ⨁⨁⨁◯ MODERATE ^a^ | **RR 0.99** (0.95 to 1.04) | 123 per 1,000 | **1 fewer per 1,000** (6 fewer to 5 more) |
| Incident Diabetes Mellitus | 42804 (4 RCTs) | ⨁⨁⨁◯ MODERATE ^b,e^ | **RR 1.10** (0.99 to 1.22) | 32 per 1,000 | **3 more per 1,000** (0 fewer to 7 more) |
| Incidence of Any Cancer | 53830 (8 RCTs) | ⨁⨁⨁◯ MODERATE ^b^ | **RR 0.97** (0.89 to 1.05) | 45 per 1,000 | **1 fewer per 1,000** (5 fewer to 2 more) |
| Myalgia | 43134 (6 RCTs) | ⨁⨁◯◯ LOW ^a,b^ | **RR 1.02** (0.88 to 1.19) | 88 per 1,000 | **2 more per 1,000** (11 fewer to 17 more) |
| Adverse Events | 33846 (6 RCTs) | ⨁⨁⨁◯ MODERATE ^a^ | **RR 0.91** (0.69 to 1.18) | 90 per 1,000 | **8 fewer per 1,000** (28 fewer to 16 more) |
| ***The risk in the intervention group** (and its 95% confidence interval) is based on the assumed risk in the comparison group and the **relative effect** of the intervention (and its 95% CI).   **CI:** Confidence interval; **RR:** Risk ratio | | | | | |
| **GRADE Working Group grades of evidence** **High certainty:** We are very confident that the true effect lies close to that of the estimate of the effect **Moderate certainty:** We are moderately confident in the effect estimate: The true effect is likely to be close to the estimate of the effect, but there is a possibility that it is substantially different **Low certainty:** Our confidence in the effect estimate is limited: The true effect may be substantially different from the estimate of the effect **Very low certainty:** We have very little confidence in the effect estimate: The true effect is likely to be substantially different from the estimate of effect | | | | | |

Supplement Table 5. Grade of evidence

a, An inconsistent effect size was present; b, no appreciable benefit or harm with statin therapy; c, an optimal information size was absent; d, the MEGA study was a significant contributor to effect size. Participants in the trial were unblinded; e, only four out of 11 trials reported the outcome.

Supplementary Material 4: Supplement Figure 1-9

**
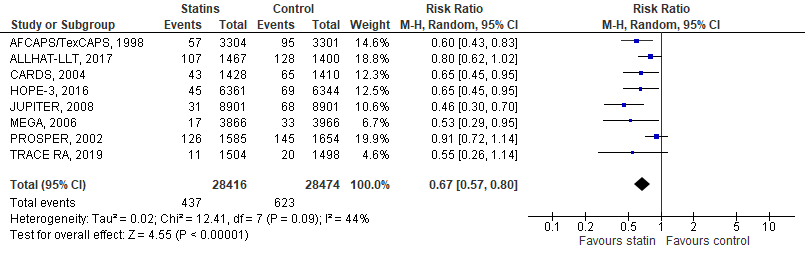
**

Supplement Figure 1. Meta-analysis of major coronary events

AFCAPS/TexCAPS, Air Force/Texas Coronary Atherosclerosis Prevention Study; ALLHAT-LLT, Antihypertensive and Lipid-Lowering Treatment to Prevent Heart Attack; CARDS, Collaborative Atorvastatin Diabetes Study; HOPE–3, Heart Outcomes Prevention Evaluation; JUPITER, Justification for the Use of Statins in Prevention: An Intervention Trial Evaluating Rosuvastatin; MEGA, Management of Elevated Cholesterol in the Primary Prevention Group of Adult Japanese; PROSPER, PROspective Study of Pravastatin in the Elderly at Risk; TRACE RA, Trial of Atorvastatin for the Primary Prevention of Cardiovascular Events in Patients with Rheumatoid Arthritis.

In the meta-analysis of major coronary events, we included events reported by trials as follows: AFCAPS/TexCAPS, HOPE-3, JUPITER, and MEGA reported any myocardial infarction (fatal and non-fatal myocardial infarction); ALLHAT-LLT reported fatal coronary heart disease and non-fatal myocardial infarction; CARDS reported fatal myocardial infarction, non-fatal MI, and other acute CHD deaths; PROSPER reported coronary heart disease deaths, and non-fatal myocardial infarction; and TRACE-RA reported non-fatal myocardial infarction.

**
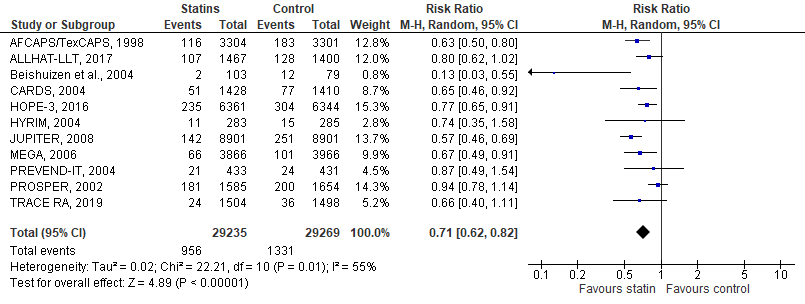
**

Supplement Figure 2. Meta-analysis of composite cardiovascular outcome

AFCAPS/TexCAPS, Air Force/Texas Coronary Atherosclerosis Prevention Study; ALLHAT-LLT, Antihypertensive and Lipid-Lowering Treatment to Prevent Heart Attack; CARDS, Collaborative Atorvastatin Diabetes Study; HOPE–3, Heart Outcomes Prevention Evaluation; HYRIM, Hypertension High Risk Management trial; JUPITER, Justification for the Use of Statins in Prevention: An Intervention Trial Evaluating Rosuvastatin; MEGA, Management of Elevated Cholesterol in the Primary Prevention Group of Adult Japanese; PREVEND IT, the Prevention of REnal and Vascular ENdstage Disease Intervention Trial; PROSPER, PROspective Study of Pravastatin in the Elderly at Risk; TRACE RA, Trial of Atorvastatin for the Primary Prevention of Cardiovascular Events in Patients with Rheumatoid Arthritis.

In the meta-analysis of composite cardiovascular outcomes, we included events reported by trials as follows: AFCAPS/TexCAPS reported fatal myocardial infarction, non-fatal myocardial infarction, unstable angina, and sudden cardiac deaths; ALLHAT-LLT reported fatal coronary heart disease and non-fatal myocardial infarction; Beishuizen et al reported unspecified cardiovascular events; CARDS reported myocardial infarction, including silent myocardial infarction, unstable angina, acute coronary heart disease deaths, and resuscitated cardiac arrest; HOPE-3 reported deaths from cardiovascular causes, non-fatal myocardial infarction or non-fatal stroke; HYRIM reported myocardial infarction, sudden death, fatal or non-fatal stroke, transient ischemic attacks, and heart failure; JUPITER reported non-fatal myocardial infarction, non-fatal stroke, cardiovascular mortality, hospitalization for unstable angina, and arterial revascularization procedures; MEGA reported first occurrence of coronary heart disease (fatal or non-fatal myocardial infarction), angina, sudden cardiac deaths, and coronary revascularization procedures; PREVEND IT reported combined incidence of cardiovascular mortality, and hospitalization for cardiovascular morbidity; PROSPER reported coronary heart disease death, non-fatal myocardial infarction, and fatal or non-fatal stroke; and TRACE RA reported non-fatal myocardial infarction, non-fatal presumed ischemic stroke, transient ischemic attack, coronary or non-coronary revascularization, cerebrovascular deaths excluding cerebral hemorrhage and non-coronary cardiac death.

**
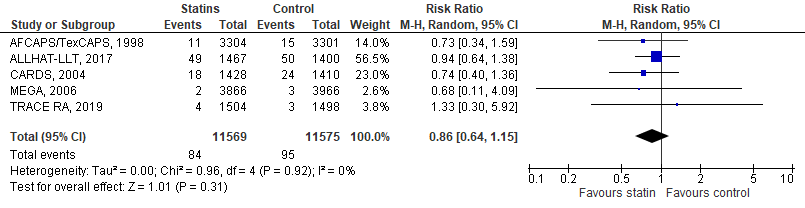
**

Supplement Figure 3. Meta-analysis of coronary heart disease mortality

AFCAPS/TexCAPS, Air Force/Texas Coronary Atherosclerosis Prevention Study; ALLHAT-LLT, Antihypertensive and Lipid-Lowering Treatment to Prevent Heart Attack; CARDS, Collaborative Atorvastatin Diabetes Study; MEGA, Management of Elevated Cholesterol in the Primary Prevention Group of Adult Japanese; TRACE RA, Trial of Atorvastatin for the Primary Prevention of Cardiovascular Events in Patients with Rheumatoid Arthritis.

In the meta-analysis of coronary heart disease mortality, we included events reported by trials as follows: AFCAPS/TexCAPS reported fatal coronary heart disease events; ALLHAT-LLT reported coronary heart disease deaths; CARDS reported occurrence of the first event as fatal myocardial infarction or other acute coronary heart disease deaths; MEGA reported fatal myocardial infarction; TRACE-RA reported coronary deaths.

**
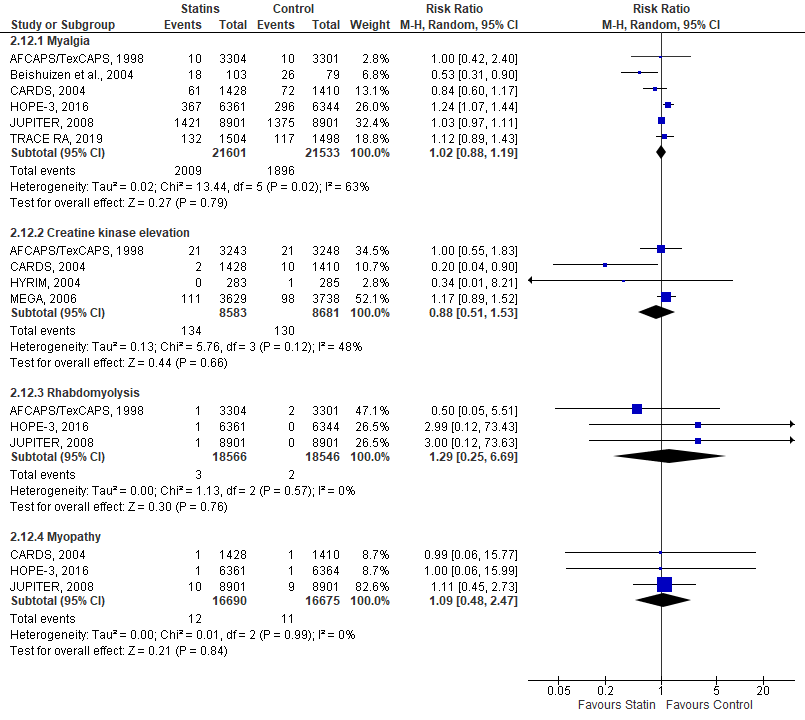
**Supplement Figure 4. Meta-analysis of muscle-related adverse events

AFCAPS/TexCAPS, Air Force/Texas Coronary Atherosclerosis Prevention Study; CARDS, Collaborative Atorvastatin Diabetes Study; HOPE–3, Heart Outcomes Prevention Evaluation; HYRIM, Hypertension High Risk Management trial; JUPITER, Justification for the Use of Statins in Prevention: An Intervention Trial Evaluating Rosuvastatin; MEGA, Management of Elevated Cholesterol in the Primary Prevention Group of Adult Japanese; TRACE RA, Trial of Atorvastatin for the Primary Prevention of Cardiovascular Events in Patients with Rheumatoid Arthritis.


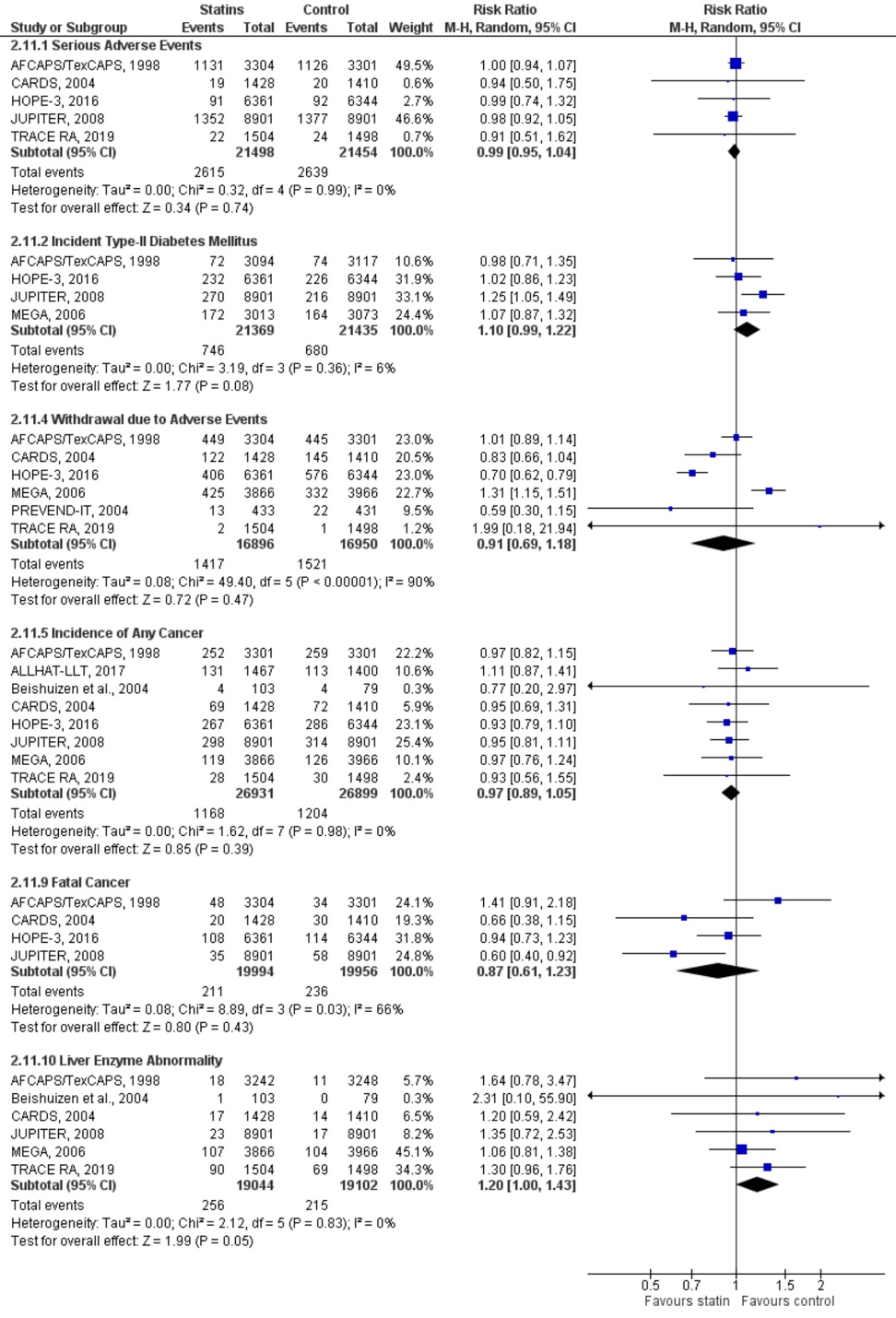


Supplement Figure 5. Meta-analysis of incidence of other adverse events

AFCAPS/TexCAPS, Air Force/Texas Coronary Atherosclerosis Prevention Study; ALLHAT-LLT, Antihypertensive and Lipid-Lowering Treatment to Prevent Heart Attack; CARDS, Collaborative Atorvastatin Diabetes Study; HOPE–3, Heart Outcomes Prevention Evaluation; JUPITER, Justification for the Use of Statins in Prevention: An Intervention Trial Evaluating Rosuvastatin; MEGA, Management of Elevated Cholesterol in the Primary Prevention Group of Adult Japanese; TRACE RA, Trial of Atorvastatin for the Primary Prevention of Cardiovascular Events in Patients with Rheumatoid Arthritis.

**
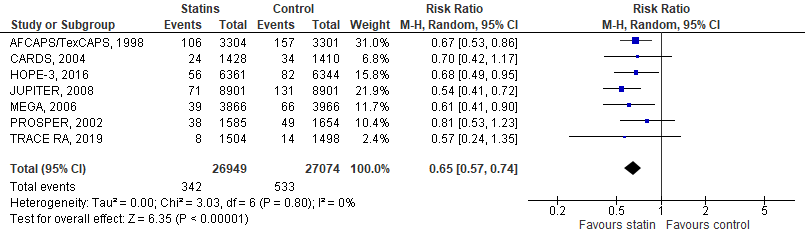
**

Supplement Figure 6. Meta-analysis of revascularizations

AFCAPS/TexCAPS, Air Force/Texas Coronary Atherosclerosis Prevention Study; CARDS, Collaborative Atorvastatin Diabetes Study; HOPE–3, Heart Outcomes Prevention Evaluation; JUPITER, Justification for the Use of Statins in Prevention: An Intervention Trial Evaluating Rosuvastatin; MEGA, Management of Elevated Cholesterol in the Primary Prevention Group of Adult Japanese; PROSPER, PROspective Study of Pravastatin in the Elderly at Risk; TRACE RA, Trial of Atorvastatin for the Primary Prevention of Cardiovascular Events in Patients with Rheumatoid Arthritis.

In the meta-analysis of revascularizations, we included events reported by trials as follows: AFCAPS/TexCAPS, HOPE-3, PROSPER reported any revascularizations; CARDS, MEGA, TRACE RA reported coronary revascularizations; JUPITER reported arterial revascularizations.

**
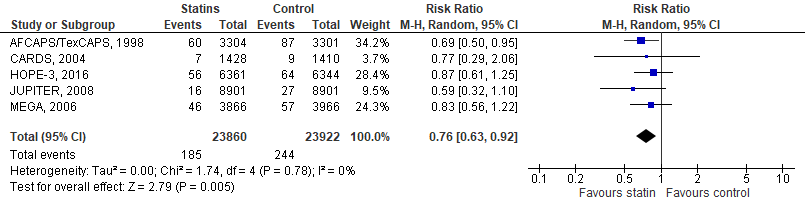
**

Supplement Figure 7. Meta-analysis of angina

AFCAPS/TexCAPS, Air Force/Texas Coronary Atherosclerosis Prevention Study; CARDS, Collaborative Atorvastatin Diabetes Study; HOPE–3, Heart Outcomes Prevention Evaluation; JUPITER, Justification for the Use of Statins in Prevention: An Intervention Trial Evaluating Rosuvastatin; MEGA, Management of Elevated Cholesterol in the Primary Prevention Group of Adult Japanese.

In the meta-analysis of angina, we included events reported by trials as follows: AFCAPS/TexCAPS, CARDS, and HOPE-3 reported unstable angina; JUPITER reported hospitalization for unstable angina; and MEGA reported any angina.

**
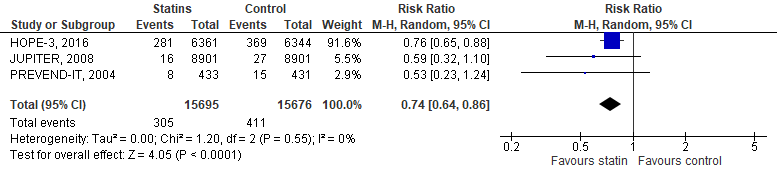
**

Supplement Figure 8. Meta-analysis of hospitalizations for cardiovascular causes

HOPE–3, Heart Outcomes Prevention Evaluation; JUPITER, Justification for the Use of Statins in Prevention: An Intervention Trial Evaluating Rosuvastatin; PREVEND IT, the Prevention of REnal and Vascular ENdstage Disease Intervention Trial.

In the meta-analysis of composite cardiovascular outcomes, we included events reported by trials as follows: (1) HOPE-3, 2016 reported hospitalizations for cardiovascular causes; (2) JUPITER, 2008 reported hospitalizations for unstable angina; and (3) PREVEND-IT reported hospitalizations for non-fatal myocardial infarction and myocardial ischemia.

**
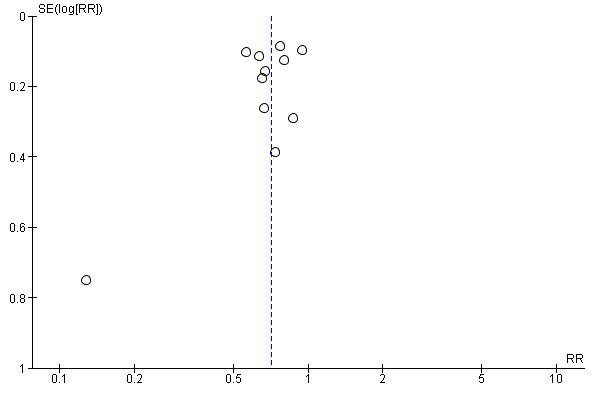
**

Supplement Figure 9. Funnel plot for composite cardiovascular outcomes
